# Recent Amendments to the Australian Privacy Act

**DOI:** 10.1007/s11673-023-10249-4

**Published:** 2023-07-11

**Authors:** Minna Paltiel

**Affiliations:** Melbourne Law School, The University of Melbourne, Parkville, Victoria 3010 Australia

**Keywords:** Privacy, *Privacy Act 1988 (Cth)*, *Privacy Legislation Amendment (Enforcement and Other Measures) Act 2022* (Cth), Privacy Act Review, Personal Information, Health Information

## Abstract

The recently passed *Privacy Legislation Amendment (Enforcement and Other Measures) Act 2022* (Cth) introduced important changes to the Australian *Privacy Act 1988* (Cth) which increase penalties for serious and repeated interferences with privacy and strengthen the investigative and enforcement powers of the Information Commissioner. The amendments were made subsequent to a number of high profile data breaches and represent the first set of changes to the *Privacy Act* following the review of the Act commenced by the Attorney-General in October 2020. The submissions made to the review emphasized the need for more effective enforcement mechanisms to increase individuals’ control over their personal information and as a form of deterrence. This article reviews the recent amendments to the *Privacy Act* and explains their effect. It comments upon the relevance of the amendments for health and medical data and other data collected in the context of healthcare, and refers to the Attorney-General’s Department’s review of the *Privacy Act* regarding other proposals relating to enforcement which have not as yet been put into effect in legislation.

## Introduction

On 28 November 2022, Parliament passed the *Privacy Legislation Amendment (Enforcement and Other Measures) Act 2022* (Cth) (“amendments”) introducing changes in the *Privacy Act 1988* (Cth) (“*Privacy Act*”). The amendments significantly increase penalties for serious and repeated interferences with privacy and strengthen the investigative and enforcement powers of the Information Commissioner (“Commissioner”).

The amendments are effectuated as part of the review of the *Privacy Act* commenced by the Attorney-General in October 2020 (“Review”). In October 2021, the Attorney-General’s Department released a comprehensive discussion paper summarizing the submissions made to the Review, setting out proposals for reform, and seeking further feedback on proposed changes to the *Privacy Act* (Australian Government 2021). The Review addresses a number of important issues bearing upon the sufficiency of *Privacy Act* regulation in the current “increasingly complex regulatory environment” to manage personal information (Australian Government 2021, 2). Included in the concerns addressed in the Review are: the *Privacy Act*’s effectiveness in protecting personal information and promoting good privacy practices, whether the Act should provide individuals a direct right of action to enforce their privacy obligations, whether a statutory tort for serious invasions of privacy should be introduced, and the effectiveness of enforcement powers and mechanisms under the *Privacy Act*. The submissions made to the Review emphasized that more effective enforcement mechanisms were necessary both to increase “individual control over privacy” and as a form of deterrence (Australian Government 2021, 8).

The amendments respond to proposals made in the Review for strengthened regulation and enforcement of *Privacy Act* protections. The changes were introduced and moved quickly through Parliament on the background of several high profile data breaches during the months of October and November 2022, including the Optus, Medibank, and MyDeal cyberattacks, and in response to what were viewed as urgent issues arising from the breaches. In his second reading speech, the Attorney-General noted the serious emotional and financial harm caused by these breaches and stated the amendments’ aim to send “a clear message that entities must take privacy, security and data protection seriously” (Commonwealth of Australia 2022, 1979).

The protections afforded under the *Privacy Act* apply to “personal information.” “Personal information” includes a subcategory of “sensitive information,” which refers to information which carries the risk of more serious harm if misused. The *Privacy Act* definition of “sensitive information” includes “health information” (*Privacy Act* s 6). “Health information” is personal information which is, amongst other things, information or opinion about health, illness, disability, or injury, and information about provision of health services, or an individual’s expressed wishes about provision of health services. It extends also to other personal information collected in the process of providing health services (*Privacy Act* s 6FA). The intended effect of the amendments therefore includes strengthened protection of individuals’ privacy in relation to their health and medical data, and their personal information collected in seeking and receiving healthcare.

The following article reviews the new amendments to the *Privacy Act* and explains their effect. It then comments upon the relevance of the amendments for health and medical data, including data held in the My Health Records system.^1^ It concludes in referring to a number of additional issues and proposals relating to enforcement raised in the Review which have not been incorporated into the current amendments.

## The Amendments

The amendments aim to enhance protection of personal information (including health information) and strengthen the enforcement and deterrence effect of the *Privacy Act*, in four ways (Commonwealth of Australia 2022, 1979). First, they increase the penalties for serious and repeated interferences with privacy. Second, they enhance the powers of the Commissioner to deal with and resolve privacy breaches. Third, they strengthen the existing Notifiable Data Breach scheme (“NDB scheme”) set out in Part IIIC of the *Privacy Act.* Fourth, they expand the information sharing powers of the Commissioner with other regulators,^2^ to enable greater cooperation and efficiency in responding to privacy risks.

### Increased Penalties for Serious and Repeated Interferences with the Privacy of an Individual

The amendments incentivize organizations to be vigilant about privacy protection by increasing penalties for serious and repeated privacy breaches under section 13G of the *Privacy Act.* Previously, the maximum penalty for a body corporate was $2.22 million. The explanatory memorandum notes that this penalty fell short of community expectations in view of the extent of financial and emotional harm caused by serious and repeated privacy breaches (Explanatory Memorandum 2022, 6). The new section 13G sets the maximum penalty for bodies corporate at an amount that is the greater of $50,000,000, three times the value of any benefit directly or indirectly attributable to the conduct constituting the breach or, if the court is unable to determine the value of that benefit, 30 per cent of the adjusted turnover of the company during the turnover period of the contravention (*Privacy Act* s 13G(3)). For a person (who is not a corporation), the amendments raise the maximum penalty for serious and repeated interferences with privacy from $444,000 to $2,500,000 (*Privacy Act* s 13G(2)). The amendments have brought these penalties in line with those imposed under the Australian Consumer Law,^3^ the *Competition and Consumer Act 2010* (Cth) and the *Australian Securities and Investments Commission Act 2001* (Cth).^4^

The new, more stringent penalties are aimed at driving businesses to implement stronger data privacy protection measures, including cybersecurity and data security safeguards. The effect of the previous low-level penalty meant that privacy breaches could be seen by companies “simply as the cost of doing business” (Commonwealth of Australia 2022a, b, 1980). The significant increase in the maximum penalty for a body corporate, which made it substantially higher than that imposed on an individual, was intended to deter breaches of privacy occurring in large companies or digital platforms for whom a $2.5 million dollar fine provided little deterrence in the context of benefits gained through the contravening conduct (Commonwealth of Australia 2022a, b, 6).

### Enhanced Enforcement Powers for the Information Commissioner

As described in the Attorney-General’s second reading speech, the amendments provide the Commissioner with a “suite of improved and new powers to resolve privacy breaches efficiently and effectively” (Commonwealth of Australia 2022, 1979).

#### Declarations, Supervision, and Audit

Part V of the *Privacy Act* deals with investigations into entities’ acts and practices which may constitute privacy breaches. The Commissioner may undertake an investigation into such acts or practices, either on its own initiative or in response to complaints. Generally, in the context of these investigations, the Commissioner may conciliate complaints, make inquiries, or may require an entity to provide information or documents, or to attend a compulsory conference (*Privacy Act* ss 40A, 42, 44, 46). (In certain circumstances, the Commissioner may transfer the matter to one of the alternative complaint bodies specified in section 50 of the *Privacy Act.*) Under section 52, a determination of the Commissioner following its investigation may result in any or all of a number of declarations, including: that an entity’s conduct constituted an interference with privacy; that the entity must refrain from continuing or repeating such conduct; that the entity take specified steps to ensure the conduct is not repeated or continued; and/or, that the entity must undertake reasonable action to redress any loss suffered by a complainant. Significantly, the amendments now permit the Commissioner to publish its determination on the Office of the Australian Information Commissioner (“OAIC”) website (*Privacy Act* s 52(5A)).

The amendments expand and strengthen the Commissioner’s ability to supervise and audit compliance with declarations. First, the amendments empower the Commissioner to require the entity to engage a qualified, independent adviser to review the acts and practices of the entity and the steps taken by the entity pursuant to the Commissioner’s declaration, and to provide a copy of its review to the Commissioner (*Privacy Act* s 52(1AAA)).

Second, the amendments empower the Commissioner to make a declaration that the entity must “prepare and publish, or otherwise communicate, a statement about the conduct …” (*Privacy Act* ss 52 (1)(b)(iia), (1A)(ba)). This means that an entity the subject of the Commissioner’s investigation, either in response to a complaint or through the Commissioner’s initiative, may now be required to prepare a statement in consultation with the Commissioner, including a description of the contravening conduct and the steps taken by the entity to ensure that it is not continued or repeated. The entity may be required to provide the statement to the complainant, or to publish it, and to verify to the Commissioner that this was done (*Privacy Act* s 52A).

The new provisions allowing the statements prepared by an entity subject to a determination, as well as the determination itself, to be published are a significant development. This change aims to keep the community informed and empowered regarding individuals’ personal information and regarding general privacy issues which may arise (Explanatory Memorandum 2022, 5). It is expected that by making this information public, individuals will have greater control over their personal information and their privacy interests.

#### Information Gathering Powers

The *Privacy Act* empowers the Commissioner to conduct assessments of an entity’s handling of personal information and whether it complies with the *Privacy Act* (including specifically, the Australian Privacy Principles (“APP”s) contained in Schedule 2 of the Privacy Act). Such assessments require the entity’s cooperation in providing information to the Commissioner. Under the new provisions, the Commissioner may require the entity to give information or produce documents within a notified timeframe (*Privacy Act* s 33C(3)). Further, the amendments to section 66 of the Act make a failure to provide information or produce a document when required under the *Privacy Act,* a civil penalty contravention (60 penalty units), and where a corporation fails to do this in a systemic or ongoing manner, it commits a criminal offence (300 penalty units) (*Privacy Act* s 66(1), (1AA)). Pursuant to the new section 80UB, the failure to provide information or documents as required under the Act is subject to an infringement notice under the *Regulatory Powers Act 2014* (Cth).

The effect of these amendments is that the Commissioner may now issue a civil penalty for minor cases of non-compliance with the requirement to provide information or documents, without having to prosecute a criminal offence or having to litigate a civil matter. This enables a more efficient process for minor contraventions, and expediates the Commissioners investigations and resolution of privacy complaints by removing delays caused by entities failure to provide information. In the case of more serious, systemic non-compliance, the Commissioner can refer the matter to the Department of Public Prosecutions (Explanatory Memorandum 2022, 7).

In addition, in line with the amendments allowing publication of determinations to keep the Australian community informed and notified, the amendments now also enable the Commissioner to publish information relating to an assessment on its website (*Privacy Act* s 33C(8)).

### *Scope of the* Privacy Act

The extraterritorial jurisdiction of the *Privacy Act* applies to entities which have an “Australian link” (*Privacy Ac*t s 5B(3)). The amendments extend the reach of the extraterritorial jurisdiction of the *Privacy Act*, by amending the definition of “Australian link” in section 5B. Previously this required that the personal information in question was collected or held by an entity in Australia before or at the time of the act in question. In the current technological environment Australians’ personal information may be processed on servers outside Australia, where the information was not directly collected from an Australian source. Previously, because of the requirement to establish an “Australian link,” the processing of personal information in these circumstances would not come under *Privacy Act* protections. However the amended s 5B(3) means that a foreign entity “carrying on business” in Australia and dealing with the personal information of Australians, will be bound by enforceable *Privacy Ac*t requirements, even if the information was not collected directly from a source within Australia (Explanatory Memorandum 2022, 2, 12–13).^5^ Although this amendment may have been directed at the practices of large, commercial digital platforms, it could also be relevant in the context of international research projects using Australian health and medical data.

### Strengthening the Notifiable Data Breach Scheme

The NDB scheme is contained in Part IIIC of the *Privacy Act.* The NDB scheme was introduced in 2016 in the wake of a number of high profile data breaches (Commonwealth of Australia 2016, 2430). In the event of a data breach involving personal information that is likely to result in serious harm, the NDB scheme requires entities to notify the Commissioner and affected individuals.

The new Division 4, introduced by the amendments, empowers the Commissioner to require an entity to produce documents or give information which the Commissioner reasonably believes are relevant to an actual or suspected data breach, or the entity’s compliance with the NDB scheme. Failure to comply with this requirement is subject to the section 66 penalties described above. The amendments also include the word “particular” with regard to the kinds of information concerned (*Privacy Act* ss 26WK(3)(c) and 26WR(4)(c)) so that the Commissioner, as well as an affected individual notified of the breach, has more specific and comprehensive knowledge of what information was compromised and the consequent risks (Explanatory Memorandum 2022, 15).

The new Division 4 operates to ensure that the Commissioner more efficiently obtains and has full knowledge of the nature of the personal information involved in a data breach, the seriousness and the scope of the breach, and the particular risks of harm flowing from the breach. This is intended to better enable the Commissioner to decide upon the action to be taken and more quickly act to minimize harms (Explanatory Memorandum 2022, 2).

### Greater Information Sharing Powers

In addition to the power to publish determinations or information relating to assessments on its website, the new section 33B authorizes the Commissioner to disclose any information obtained through exercising its powers or performing is functions, if doing so is in the public interest. The statute provides factors which must be considered in determining when such disclosure is in the public interest, including the rights and interests of any complainant or the entity itself, or whether any individual’s personal information or confidential commercial information will be disclosed. The explanatory memorandum describes this change as aiming to ensure that “Australians are informed about privacy issues and to reassure the community that the OAIC is discharging its duties … ” (Explanatory Memorandum 2022, 17).

The amendments also expand the Commissioner’s ability to share information with enforcement bodies, alternate complaints bodies, and other regulators whose functions include privacy protection of individuals (*Privacy Ac*t s 33A). Such sharing is at the Commissioner’s discretion and is subject to limitations of reasonability, necessity, and proportionality set out in the Act. (The Commissioner is generally restricted in disclosing information by section 29 of the *Australian Information Commissioner Act 2010* (Cth).) Prior to the amendments, there were only limited circumstances under which the Commissioner was authorized to share information with other authorities. By extending the Commissioner’s information sharing ability, the amendments aim to improve cooperation and increase capacity to efficiently act to minimize harms caused by privacy breaches (Explanatory Memorandum 2022, 8).

The new provisions permitting the Commissioner to publish certain information are in effect “privacy limiting” provisions, representing legal exceptions to the prohibition of disclosing personal information for purposes other than those to which an individual consented (Explanatory Memorandum 2022, 6, 9). APP 6 prohibits use or disclosure of personal information other than with the individual’s consent unless under specified exceptions. These exceptions include the use or disclosure of personal information where authorized by law (APP 6.2(b)). The information sharing provision in sections 33A, and authority of the Commissioner to publish determinations and information relating to its investigations and assessments in sections 33B and 33C(8) operate as new instances of authorization under law to disclose personal information. Importantly, these limitations of privacy are subject to statutory safeguards. They are permitted as being “reasonable, necessary and proportionate means” of achieving better and more efficient cooperation between regulatory and law enforcement bodies in enforcing *Privacy Act* compliance, and in ensuring that Australians are informed when their privacy may have been compromised and enabled to act to protect their personal information (Explanatory Memorandum 2022, 9).

## Relevance for Health and Medical Data

The recent amendments to the *Privacy Act* have important implications for treatment of medical and health data. Because health information is protected by the Act, what could be described as the “longer and stronger arm” provided the Commissioner under the amendments will potentially translate to greater supervision, auditing, and accountability with regard to medical and health data. In addition, the provisions permitting the publication of information pertaining to privacy violations would mean greater transparency, so that as with all personal information, Australian individuals will be better informed and more able to determine the uses and protect against any misuses of their health and medical information. Because of how health information is defined in the *Privacy Act,* this also applies with regard to any personal information collected, used, and disclosed in the process of seeking or receiving healthcare.

The amendments are also relevant regarding the information held in the My Health Records system. A contravention of the *My Health Records Act* 2021 (Cth) (*“MHR Act”*) in connection with information held in a person’s My Health Record is also an interference with privacy for the purposes of the *Privacy Act* (*MHR Act* s 73). Such a breach may be the subject of a complaint or of an investigation initiated by the Commissioner under the *Privacy Act*. The amendments pertaining to the Commissioner’s investigative powers, including the ability to publish information relating to investigations, would apply in this case. In addition, certain conduct could contravene both an *MHR Act* penalty provision and a *Privacy Act* penalty provision (Commonwealth of Australia 2016, s 14). Where a contravention of the *MHR Act* is also a “serious and repeated” interference with privacy under the *Privacy Act*, the breaching entity may be subject to a section 13G penalty, and the amendments would come into play. As such, the amendments affecting the powers of the Commissioner in these function impact upon information in a My Health Record as they do upon personal information under the *Privacy Act*.^6^

## The Amendments and the Privacy Act Review

The current amendments are only the first of the proposed reforms of the *Privacy Act* emerging from the Review. The amendments to a large extent reflect proposals made in the Review pertaining to regulation and enforcement, however, there are some proposed changes relating to enforcement which have not been put into effect in the current amendments and which are worth noting. One such proposal was to address less serious breaches of privacy through creating “tiers of civil penalties,” including a mid-tier civil penalty with a lower maximum penalty than that for serious and repeated breaches of privacy (Australian Government 2021, 171–175). This proposal would enable some regulatory response even when the breach does not meet the threshold of “serious and repeated” interferences with privacy. The suggestion to clarify the threshold of what constitutes “serious and repeated interferences with privacy” within the Act (Australian Government 2021, 175–176) has likewise as yet not been addressed.

The Review has also raised the proposal to create a direct right of action for interference with privacy, available to any individual or group of individuals (Australian Government 2021, 184–188). This would mean that after a claimant had lodged a complaint with the OAIC, and where the matter was not conciliated, or the complainant chose not to pursue conciliation, that complainant could initiate Court action. The summary of submissions noted various models for the introduction of a statutory tort of privacy. It is of note that a statutory tort of privacy has been twice previously recommended by the Australian Law Reform Commission (Australian Law Reform Commission 2014, 61–71; Australian Law Reform Commission 2008, 2556–2584). However, to date, the Australian legislature has resisted this move.

One proposal of the Review which is of relevance for health and medical data is regarding whether there should be a greater degree of consistency between other legislation relating to privacy, with which the *Privacy Act* interacts. A number of submissions to the Review called for creating specific legislation imposing more stringent protections where a scheme (such as the My Health Records scheme) deals with sensitive information carrying with it specific risks and concerns. The submissions supported legislating additional obligations reflecting community expectations in these cases and associated requirements for greater oversight and enforcement powers on the part of the Commissioner (Australian Government 2021, 209). This proposal has not to date been taken up by the legislature. Should it be in the future, it would further heighten protection of health and medical data, which are sensitive information.

## Concluding Comments

The recent amendments to the *Privacy Act* represent the first step in updating the *Privacy Act* as an effective regulatory tool addressing data practices in today’s technological environment. Recent large scale data breaches have shown that the privacy of individuals’ personal information could be compromised in circumstances where little can be done to enforce compliance or redress harm. The amendments aim to strengthen enforcement and regulation, and to better inform and empower the Australian community. Potentially the amendments will in fact incentivize better privacy protection practices and security measures in entities dealing with any personal information, including health information, and function to empower Australian individuals to take steps and make choices to protect their personal information. The impact of the current changes is now to be seen, and it is expected that further changes and amendments to the *Privacy Act* will follow.

## Footnotes

1 The “My Health Records” system (“MHR system”) is a voluntary national digital health record system governed by the *My Health Records Act *2012 (Cth) (“*MHR Act*”). About 90 per cent of Australians participate in the MHR system making the MHR database a very substantial repository of Australian health information.

2 This article confines itself to changes in the *Privacy Act,* however it is of note that the amendments also operate to amend the *Australian Communications and Media Authority Act 2005* (Cth) regarding that authority’s information sharing powers.

3 The Australian Consumer Law is found in the *Competition and Consumer Act 2010* (Cth), sch 2.

4 This Act was also recently amended by the *Treasury Laws Amendment (More Competition, Better Prices) Bill 2022* (Cth) which was passed on 27 October 2022.

5 The case of *Australian Information Commissioner v Facebook Inc (No 2)* [2020] FCA 1307 addressed this issue in the superseded section 5B definition of “Australian Link” and the practical difficulties in establishing that a foreign entity collected personal information directly from Australia.

6 Note that the Commissioner has investigative powers and function under the MHR Act in addition to its power under the *Privacy Act* and has the power to do anything necessary or convenient to fulfill these functions (MHR Act, s 73(4)).
